# High-definition transcranial direct current stimulation of the left middle temporal complex does not affect visual motion perception learning

**DOI:** 10.3389/fnins.2022.988590

**Published:** 2022-09-01

**Authors:** Di Wu, Yifan Wang, Na Liu, Panhui Wang, Kewei Sun, Wei Xiao

**Affiliations:** ^1^Department of Medical Psychology, Air Force Medical University, Xi’an, China; ^2^Department of Nursing, Air Force Medical University, Xi’an, China

**Keywords:** direct current stimulation (tDCS), brain stimulation, human middle temporal complex (hMT+), visual perceptual learning (VPL), motion direction discrimination

## Abstract

Visual perceptual learning (VPL) refers to the improvement in visual perceptual abilities through training and has potential implications for clinical populations. However, improvements in perceptual learning often require hundreds or thousands of trials over weeks to months to attain, limiting its practical application. Transcranial direct current stimulation (tDCS) could potentially facilitate perceptual learning, but the results are inconsistent thus far. Thus, this research investigated the effect of tDCS over the left human middle temporal complex (hMT+) on learning to discriminate visual motion direction. Twenty-seven participants were randomly assigned to the anodal, cathodal and sham tDCS groups. Before and after training, the thresholds of motion direction discrimination were assessed in one trained condition and three untrained conditions. Participants were trained over 5 consecutive days while receiving 4 × 1 ring high-definition tDCS (HD-tDCS) over the left hMT+. The results showed that the threshold of motion direction discrimination significantly decreased after training. However, no obvious differences in the indicators of perceptual learning, such as the magnitude of improvement, transfer indexes, and learning curves, were noted among the three groups. The current study did not provide evidence of a beneficial effect of tDCS on VPL. Further research should explore the impact of the learning task characteristics, number of training sessions and the sequence of stimulation.

## Introduction

Visual perceptual learning (VPL) refers to training-induced improvements in human visual perceptual abilities, ranging from simple visual feature discrimination to complex object recognition (Dosher and Lu, 2017; Zhang et al., 2018; Wu et al., 2021a). VPL can be considered a manifestation of brain plasticity; thus, investigating its neural mechanisms will improve understanding of the neural plasticity of the adult brain (Kawato et al., 2014). Over the past decades, researchers have mainly focused on two important facets of the neural mechanisms underlying VPL: the location (i.e., the cortical loci) and form of the plasticity-related changes. In addition, an increasing number of studies have translated VPL findings to clinical applications (Lu et al., 2016). VPL has been used to treat patients with various types of vision loss, such as cortical blindness (Huxlin et al., 2009; Das et al., 2014; Herpich et al., 2019), amblyopia (Polat et al., 2004; Huang et al., 2008; Levi and Li, 2009; Astle et al., 2011; Barollo et al., 2017), macular degeneration (Astle et al., 2015; Maniglia et al., 2016), myopia (Tan and Fong, 2008; Casco et al., 2014; Yan et al., 2015), visual field defects (Casco et al., 2018) and presbyopia (Sterkin et al., 2018). However, adequate performance enhancements usually require substantial training (Herpich et al., 2019), which limits the practical applications of VPL. Methods for maximizing the effects of training (e.g., increasing the magnitude of improvement, learning speed, and generation of trained skills, or extending the persistent effects of learning) have attracted increasing interest.

Research has shown that noninvasive transcranial electrical stimulation (tES) not only directly improves visual perception (Ding et al., 2016; Bocci et al., 2018) but also promotes perceptual learning when coupled with behavioral training, such as transcranial direct current stimulation (tDCS; Pirulli et al., 2013; Karlaftis et al., 2021), transcranial alternating current stimulation (tACS; He et al., 2022) and random noise stimulation (RNS; Contemori et al., 2019), benefiting for clinical patients such as amblyopia (Spiegel et al., 2013; Campana et al., 2014; Moret et al., 2018), mild myopia (Camilleri et al., 2014, 2016) and hemianopia after occipital stroke damage (Plow et al., 2011, 2012). Among tES techniques, tDCS is particularly attractive due to its low cost and portability (Reinhart et al., 2016). tDCS transiently modulates cortical excitability by altering the membrane potential of neurons (Stagg and Nitsche, 2011; Stagg et al., 2011). In general, the cortical excitability is increased by the anodal electrode and decreased by the cathodal electrode (Parkin et al., 2015; Woods et al., 2016). In the first study, anodal tDCS over the extrastriate visual cortical area (V5) or primary motor cortex (M1) was found to significantly increase learning in the early phase, but no significant effect was found for cathodal stimulation (Antal et al., 2004a). Additionally, Sczesny-Kaiser et al. applied tDCS over the primary visual cortex (V1) while participants learned how to perform a visual orientation-discrimination task. These researchers found that anodal tDCS improved VPL and increased cortical excitability. Recently, Karlaftis et al. (2021) applied tDCS over the right occipito-temporal cortex (OCT) while participants trained on a signal-in-noise task and found that anodal tDCS boosted learning by reducing GABA+ levels and by altering local processing in the visual cortex as well as functional connectivity between visual and posterior parietal areas compared to sham tDCS.

However, other tDCS studies on visual learning have reported varying results. Some studies did not find that tDCS influenced visual learning when applied while participants trained on orientation discrimination tasks (Fertonani et al., 2011; Pirulli et al., 2013; Larcombe et al., 2018). Additionally, overnight consolidation of visual learning was blocked by anodal tDCS applied while participants trained on a contrast detection task (Peters et al., 2013). Thus, further empirical data in this area are needed. The current study has several improvements in experimental design compared to previous studies. Psychophysical research typically describes VPL according to various aspects, namely, the magnitude of learning, learning curves, specificity and transference; these aspects reveal possible changes in visual processing in the brain regions associated with learning in greater detail. However, previous studies have mainly focused on the effect of tDCS on the magnitude of learning, rarely incorporating the other indicators. Therefore, this study comprehensively examined multiple psychophysical indicators, including the magnitude of improvement, learning curve and transfer index, to elucidate the relationship between tDCS and VPL.

Coherent motion is frequently employed in studies regarding the neuromodulation of visual perception (Olma et al., 2013; Battaglini et al., 2017, 2020) given that the human middle temporal complex (hMT+) has been confirmed to play a specialized role in visual motion perception with multiple techniques, such as lesions (Newsome and Pare, 1988), electrophysiology (Britten et al., 1992), functional magnetic resonance imaging (fMRI; Chen et al., 2016, 2017) and electrical stimulation (Antal et al., 2004b; Pavan et al., 2019; Wu et al., 2020). For example, the visual area V1 receives visual information from the eye; thus, cortical blindness is induced by any damage to this region. However, many patients whose V1 is damaged show activity in the hMT+ (Bridge et al., 2010; Sara et al., 2015), and some still detect moving stimuli (which are processed in this area) via a phenomenon termed blindsight (Silvanto et al., 2005).

**FIGURE 1:**
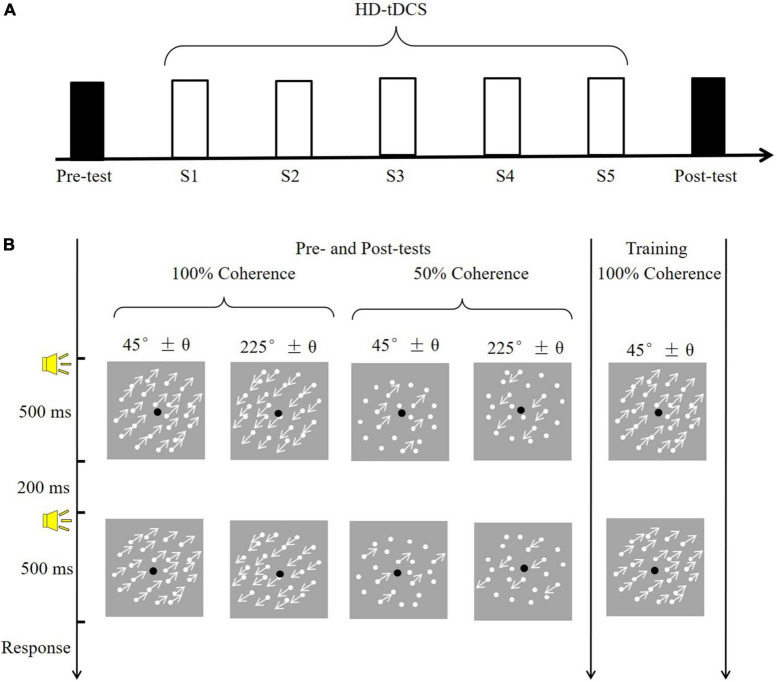
The experimental procedure and task. **(A)** A diagram of the experimental procedure. Black rectangles indicate to the pre- and post-training assessments (conducted without tDCS); white rectangles (S1–S5) indicate training sessions with simultaneous tDCS. **(B)** Example of a trial. The pre- and post-training assessments determined motion direction discrimination thresholds in four conditions, varying in coherence (100 vs. 50%) and motion direction (45° vs. 225°). The training session consisted of one condition: 100% coherence and a 225° motion direction.

Here, we tested whether the application of high-definition tDCS (HD-tDCS) over the hMT+ during training increased VPL of motion direction discrimination. HD-tDCS differs from the conventional approach in that it employs small electrodes instead of the two large sponge electrodes and thus can target more specific brain regions (Dmochowski et al., 2011). The 4 × 1 ring configuration is a typical and frequently used montage for HD-tDCS; it involves a central electrode placed over the target region, and four return electrodes placed around it in a ring-like configuration (Villamar et al., 2013). In the current study, motion direction discrimination thresholds were assessed without tDCS before (Pre) and two days after (Post) training. The pre- and post-training assessments consisted of varying stimulus types (trained: 100% coherence; untrained: 50% coherence) and motion direction (trained: 225°; untrained: 45°). Participants received five consecutive days of training, during which they simultaneously experienced anodal, cathodal or sham 4 × 1 ring HD-tDCS over the hMT+ (Figure 1A).

## Materials and methods

### Participants

Twenty-seven participants (20 males) with normal or corrected-to-normal visual acuity (VA) and a mean age of 20.5 ± 0.9 years were randomly divided into the anodal (*n* = 9), cathodal (*n* = 9) or sham (*n* = 9) tDCS conditions. No significant differences among the three groups regarding age (*p* = 0.430) or VA (*p* = 0.830) were noted. None of the participants had previous experiences with various visual perception experiments; they were blinded to the objectives of the study. The participants provided written informed consent, and the study was approved by the local Research Ethics Committee and adhered to the principles of the Declaration of Helsinki.

### Stimuli

As shown in Figure 1B, visual stimuli consisted of 400 white moving dots (0.18° in diameter), which were presented against a gray background (mean luminance: 26 cd/m^2^). The dots were randomly positioned within the round window (8° in diameter) with a moving velocity of 10°/s. The density of dots was remained constant (7.96 dots/deg^2^) through substitution with new dots at different, randomly selected locations within the window once the dots moved outside the window. In the 100% motion coherence condition, all dots moved in the same direction. In the 50% motion coherence condition, 50% of the dots served as signals (moved in the same direction), and the remainder served as noise (randomly moving direction). The movement direction of the dots was fixed at 45° (upper right of the window) or 225° (bottom left of the window).

All experimental procedures were completed in a quiet, dark room in which participants were seated in front of a computer screen. The experimental environment was kept constant in all sessions. A gamma-corrected 60 cm × 34 cm monitor was used to display the stimuli with 1,920 × 1,080 pixels spatial resolution and 85 Hz refresh rate using a computer running MATLAB (MathWorks, Natick, MA, United States) and PsychToolbox extensions. Participants binocularly viewed the displays from 75 cm away, with their head stabilized by a chinrest and headrest; the displays covered 6.84° × 3.89° of their visual field. For subjects with corrected-to-normal vision, normal VA was ensured by optical correction.

### Procedure

All observers had to complete the motion direction discrimination tests before and two-day after training. The post-test was conducted two-day after training because at least 48 h of time interval was frequently used in previous studies to limit potential carryover effects of tDCS (Wu et al., 2021b). The tests without the tDCS effect contribute to obtaining the pure improvement of VPL given that the tDCS itself could directly benefit the visual motion perception (Wu et al., 2020). During the test phase (Figure 1A), participants completed four conditions, each containing 100 trials, at two coherence levels (100 and 50%) and two motion directions (approximately 45° and 225°). The four conditions were counterbalanced and each lasted approximately 5 min. In a trial, two 500-ms visual stimuli (45° and 45 ± θ°; 225° and 225 ± θ°) were randomly displayed and separated by a 200-ms blank screen. A brief tone sounded at the beginning of each stimulus. A two-alternative forced-choice (2-AFC) response was made to judge the direction of the second visual stimulus relative to the first (i.e., clockwise or counterclockwise). A brief tone sounded after each response, regardless of accuracy. Participants adequately practiced before the formal experiment to become familiar with the tasks.

The threshold (θ°) varied by trial and was controlled by an adaptive three-down one-up staircase method to assess the thresholds of motion direction discrimination, converging to an accuracy rate of 79.4%. Specifically, this method decreased θ_*n*_ by 10% (multiplied θ_*n*–1_ by 0.9) after every three consecutive correct responses and increased θ_*n*_ by 10% after every incorrect response. Reversals were recorded when the direction of θ change shifted from increasing to decreasing or vice versa and excluded the first four or five reversals if the total number of reversals was even or odd, respectively. The remaining reversals were averaged to calculate the threshold.

During the training phase, each subject performed a motion direction discrimination task with 100% coherency and a motion direction of approximately 225°. Training sessions took place daily over five consecutive days, and the trained direction and direction were fixed for all sessions. A brief tone sounded only after a correct response. To ensure that training and application of tDCS occurred simultaneously, each training session consisted of 5 blocks of 80 trials, for a total duration of approximately 18.3 ± 0.7 min, which was shorter than the duration of stimulation (20 min). Participants were allowed to rest between blocks and initiated the next block when they were ready. The time of day and experimental environment were kept constant in all sessions. During the middle of the training session, participants were asked to report tDCS-induced sensations. Participants were specifically asked the following question: What is your sensation of the stimulation region? including any sensations such as burning, itching, tingling, pain and so on. Sensation intensity was evaluated on a 10-point scale as follows: 0 = none, 10 = strong and intolerability.

### Transcranial direct current stimulation

**FIGURE 2:**
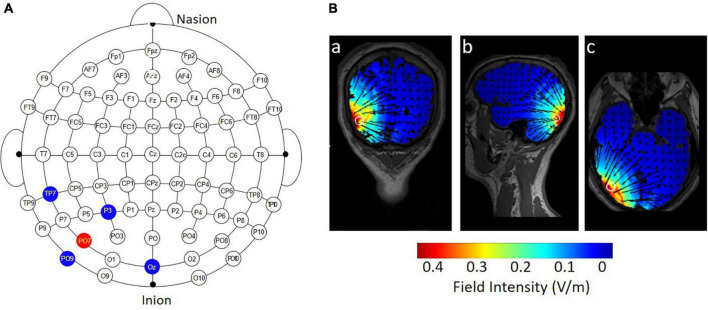
Electrode montage and simulated distribution of the electrical field during 4 × 1 HD-tDCS. **(A)** The red point represents the central electrode, placed over PO7; the blue points represent the four return electrodes, placed over P3, OZ, TP7, and PO9. **(B)** HD-Explore software was used to model the field intensity and current flow for anodal HD-tDCS: (a) coronal view, (b) sagittal view, and (c) axial view.

The majority of previous studies applied conventional 1 × 1 tDCS over the hMT+. In this method, the active electrode is placed approximately 3–4 cm above the mastoid-inion line and 6–7 cm left or right of the midline in the sagittal plane; the return electrode is placed at the vertex (Antal et al., 2012; Larcombe et al., 2018). To apply specific stimulation to the hMT+, a 4 × 1 ring HD-tDCS montage (Soterix Medical, NY, United States) was administered (Figure 2A). Zito et al. (2015) applied the HD-tDCS over the right hMT+. The electrode montage was the same and the region of interest was in the left hemisphere. Specifically, the central electrode was placed at PO7 and four return electrodes were placed at a distance of approximately 5 cm from the central electrode; their locations corresponded to P3, OZ, TP7, and PO9 (in the 10–10 standard EEG system). For anodal tDCS, the anode was placed on PO7, delivering a 20-min 1.5 mA DC (fade in/out: 30 s). The return current was equally divided through the four remaining electrodes. Conductive gel was injected into the electrode casings (1 cm in diameter) to increase conductivity and reduce impedance (<5 kΩ). For sham tDCS, the current was ramped up over 30 s at the beginning and ramped down over 30 s, and current during the 20-min middle period was 0 mA. As shown in Figure 2B, the current flow of anodal tDCS was depicted using HD-Explore software (Soterix Medical Inc., New York).

Given that the stimulator was operated by an experimenter, he/she was unblinded to whether participants were receiving anodal or sham stimulation. However, this experimenter was blinded to the purpose and the experimental design of current study.

### Data analysis

To obtain the learning curves, a power function was used


(1) $$C\,\left(t\right)=\left(C_{0}-\iota \right)\times t^{-\rho }+\iota$$

where *C_0* is the initial threshold, *t* is the training session number, ρ is the learning rate, and ι is the asymptotic line.

A nonlinear least squares method, implemented in MATLAB (MathWorks, Natick, MA, United States), was used to minimize the sum of squared differences between the model predictions and measured values. The goodness of fit was estimated by


(2) $$r^{2}=1.0-\frac{\sum {\left({\text{y}}_{m\,e\,a\,s\,u\,r\,e\,d}-{\text{y}}_{p\,r\,e\,d\,i\,c\,t\,e\,d}\right)}^{2}}{\sum {\text{[y}}_{m\,e\,a\,s\,u\,r\,e\,d}-\mathrm{mean}\left(y_{m\,e\,a\,s\,u\,r\,e\,d}\right)]{}^{2}}$$

where y_*measured*_ and y_*predicted*_ represent the measured and predicted values, respectively, and *mean*(y_*measured*_) represents the mean of all the measured values.

We compared the learning curves among the three experimental conditions (anodal, cathodal and sham) in a nested-model testing framework. An *F* test was used to compare the nested models:


(3) $$F\,\left(d\,f_{1},d\,f_{2}\right)=\frac{\left(r_{f\,u\,l\,l}^{2}-r_{r\,e\,d\,u\,c\,e\,d}^{2}\right)/d\,f_{1}}{\left(1-r_{f\,u\,l\,l}^{2}\right)/d\,f_{2}}$$

where *df*_1_=*k*_*full*_−*k*_*reduced*_, *df*_2_=*N*−*k*_full_, *k*_*full*_ and *k*_*reduced*_ are the numbers of parameters of the full and reduced models, and *N* is the number of data points. The best-fit model was defined as the model that was statistically equivalent to other models and had minimum parameters.

The transfer index was calculated as the magnitude of improvement in the untrained direction divided by the magnitude of improvement in the trained direction (Zhang et al., 2018).

In addition to the frequentist statistical approaches, a Bayesian repeated measures ANOVA was performed with the opensource software package JASP. Bayesian analyses permit a test of the relative strength of evidence for the null hypothesis (H0: no effect of tDCS stimulation group) versus the alternative hypothesis (H1: change in behavior as a result of tDCS condition). The one-way Bayesian ANOVA on transfer indexes, slope and intercept, and two-way Bayesian ANOVA on pre- and post-training performance were separately performed in JASP.

## Results

### Pre- and post-training performance

**FIGURE 3:**
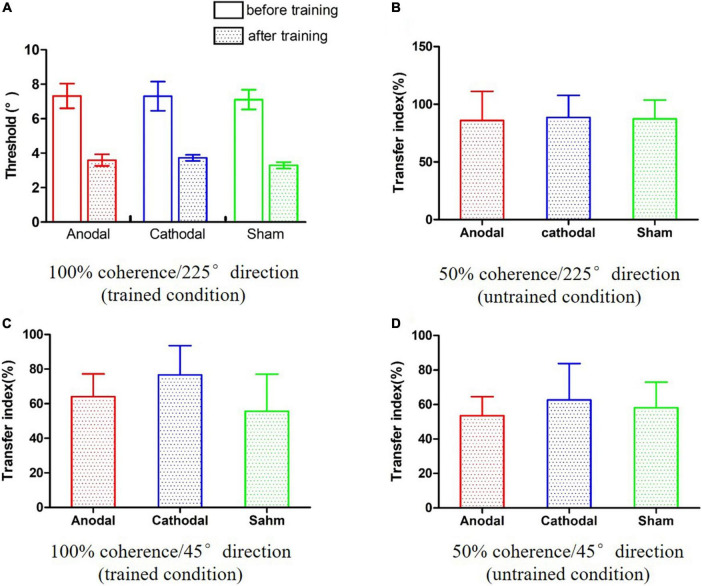
The results of the pre- and post-training assessments. **(A)** Improvements in the trained condition. **(B–D)** The transfer indexes in three untrained conditions.

A two-way analysis of variance (ANOVA) was conducted incorporating the effects of the 2 time points (pre and post) and 3 conditions (anodal, cathodal and sham) on the motion direction discrimination threshold. As shown in Figure 3A, there was a significant main effect of time, *F*(1,24) = 101.16, *p* < 0.001, η^2^ = 0.81. However, the main effect of condition and the time point × condition interaction were not significant, *F*s < 1, indicating that neither anodal nor cathodal tDCS significantly influenced performance on the motion direction discrimination task.

To further interpretate of the null effect of tDCS, a two-way Bayesian ANOVA was also performed. The consistent pattern of results was found across both frequentist and Bayesian analyses. The Bayes factor for the effect of active tDCS condition (alternative hypothesis H1: significant difference among anodal, cathodal and sham tDCS) was less than hundredth (BF_10_ = 3.00e-12), providing extreme evidence for supporting the null hypothesis.

### Effect of transcranial direct current stimulation on learning transference

The transfer indexes were calculated for three conditions: 50% coherence/225° direction, 100% coherence/45° direction, and 50% coherence/45° direction. We conducted one-way ANOVAs and one-way Bayesian ANOVAs on the transfer indexes of the three conditions. The pattern of results was consistent across both frequentist and Bayesian analyses. There were no significant main effects of conditions in the 50% coherence/225° direction condition (Figure 3B), *F*(2,24) < 0.001, *p* = 0.996, η^2^ < 0.01, BF_10_ = 0.227; BF_01_ = 4.408; 100% coherence/45° direction condition (Figure 3C), *F*(2,24) = 0.37, *p* = 0.697, η^2^ = 0.03, BF_10_ = 0.295; BF_01_ = 3.510; or 50% coherence/45° direction condition (Figure 3D), *F*(2,24) = 0.08, *p* = 0.925, η^2^ = 0.01, BF_10_ = 0.238; BF_01_ = 4.207. The Bayesian ANOVAs provided anecdotal evidence in favor of the null hypothesis. Taken together, these results suggest that tDCS did not influence learning transference to untrained values of coherence and direction.

### Effect of transcranial direct current stimulation on learning over multiple sessions

**FIGURE 4:**
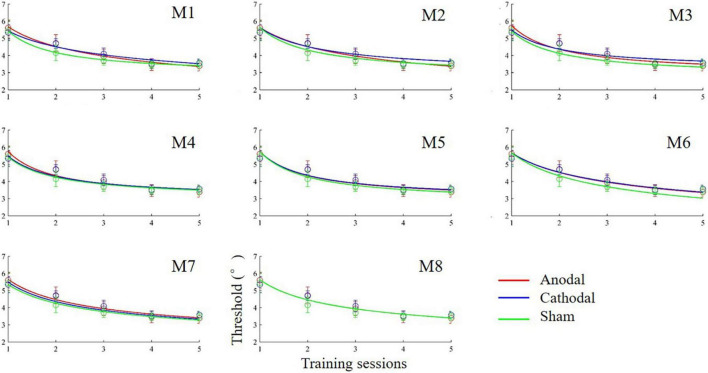
Learning curves (discrimination threshold as a function of training session) fit by power functions in different models.

The anodal, cathodal and sham learning curves were estimated by a power function with a total of nine parameters. That is, each learning curve had three parameters: the initial threshold (*C_0*), learning rate (ρ) and asymptotic line (ι). Thus, eight models were developed by setting some parameters to be equivalent (Table 1). Specifically, we constructed 8 models: a full 9-parameter model (M1) with independent *C_0*, ρ andι values; reduced 7-parameter models with identical *C_0*, ρ orι values (M2, M3 and M4); reduced 5-parameter models with identical *C_0*/ρ (M5), *C_0*/ι (M6) and ρ/ι (M7) values; and a reduced 3-parameter model with identical *C_0*, ρ andι values (M8). The eighth model (M8) was the most reduced (simplest) model and was statistically equivalent to the other models (all *p*s > 0.100; Figure 4). Thus, M8, which held all parameters equal across the three conditions provided the best fit, suggesting that tDCS did not significantly influence learning over multiple training sessions.

**TABLE 1:** Comparison of model fits to the learning curves.

	M2	M3	M4	M5	M6	M7	M8	r^2^(%)	Parameters
M1	0.080	0.037	0.137	0.094	0.010	0.205	0.266	98.03	3*C*_0_, 3ρ, 3ι
M2				0.279	0.293	0.917	0.848	95.43	1*C*_0_, 3ρ, 3ι
M3				0.787	0.827	1	1	94.08	3*C*_0_, 1ρ, 3ι
M4				0.136	0.143	0.448	0.562	96.18	3*C*_0_, 3ρ, 1ι
M5							1	93.71	1*C*_0_, 1ρ, 3ι
M6							1	93.79	1*C*_0_, 3ρ, 1ι
M7							0.514	95.33	3*C*_0_, 1ρ,1ι
M8								94.67	1*C*_0_, 1ρ, 1ι

Columns 2 to 8 display the p values of statistical comparisons between different models.

The model parameters are shown in the right column.

### Effect of transcranial direct current stimulation on learning within a session

The above analyses did not find a significant effect of tDCS on learning curves over five days of consecutive sessions. The next analysis focused on learning curves within each session (online effect; Reis et al., 2009). We averaged thresholds within the same block for the five sessions. Based on the observed trends, a linear regression model was used to fit the within-session learning curves as a function of the block. The slope and intercept of the learning curves were estimated with a linear least squares method. One-way ANOVAs and one-way Bayesian ANOVAs were conducted on the slope and intercept. The ANOVA on the slope did not find a significant difference among the anodal, cathodal and sham groups, *F*(2,24) = 0.81, *p* = 0.457, η^2^ = 0.06, BF_10_ = 0.375; BF_01_ = 2.668. Similarly, no obvious difference in intercept was found among the three groups, *F*(2,24) = 0.05, *p* = 0.952, η^2^ = 0.01, BF_10_ = 0.233; BF_01_ = 4.283. The Bayesian ANOVAs provided anecdotal (slope) or moderate (intercept) evidence in favor of the null hypothesis. These results indicated that tDCS had no effect on learning within a session. slope.

### Sensations induced by transcranial direct current stimulation

**FIGURE 5:**
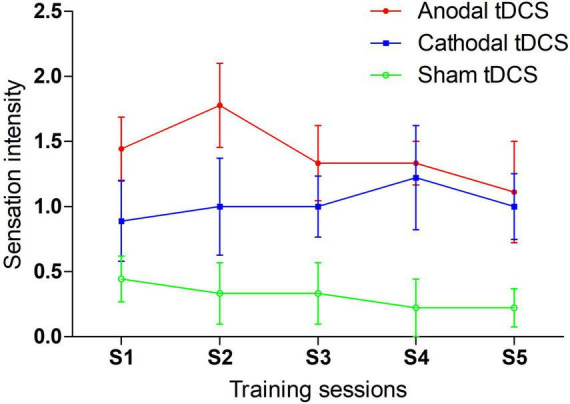
tDCS induced sensation during 5-day training sessions.

Each participant completed a question at the middle point of each training session. The results are reported in Figure 5. We compared the sensation intensity among anodal, cathodal and sham tDCS conditions with one-way ANOVAs. The main effect was significant or marginally significant for each session. The *p* values from sessions 1 to 5 were 0.030, 0.013, 0.032, 0.019, and 0.090, respectively. *Post-hoc* LSD analyses were further conducted for each session. The anodal tDCS induced significantly greater sensation intensity than sham tDCS for all training sessions: session 1 (*p* = 0.009), session 2 (*p* = 0.004), session 3 (*p* = 0.010), session 4 (*p* = 0.010), and session 5 (*p* = 0.036). Additionally, *p* values of difference in sensation intensity between cathodal and sham tDCS were 0.218, 0.149, 0.076, 0.019, and 0.109 from sessions 1 to 5, respectively. No significant differences in sensation intensity were noted between anodal and cathodal tDCS for all sessions [session 1 (*p* = 0.127), session 3 (*p* = 0.364), session 4 (*p* = 0.783) and session 5 (*p* = 0.584)] with the exception of session 2, which was marginal significant (*p* = 0.094). In general, the anodal tDCS-induced sensations were perceived more strongly than sham-induced sensations; moreover, anodal tDCS was indistinguishable from cathodal tDCS, especially during the late period of training.

## Discussion

After five consecutive days of training, the motion direction discrimination thresholds significantly decreased. However, 4 × 1 ring HD-tDCS applied on the left hMT+ did not affect VPL in terms of the magnitude of improvement, learning curve or transference.

There are three possible explanations for the failure to identify an effect of tDCS on VPL. First, there may have been no room for improvements on the training task. Coherent motion tasks are frequently employed to investigate visual perception and its neural mechanisms since it has a relatively distinct brain region (i.e., the hMT+). The coherent motion task has many variations, depending on the research topic. For example, the motion direction discrimination task in this study was derived from a previous study conducted by Chen et al. (2016, 2017). Their task involved discriminating the global direction of moving dots. Specifically, the moving dots were presented twice, and participants then determined the minimum angle of the two directions of moving dots. Obviously, that task required two cognitive processes: identifying the direction of motion against a noisy background and then discriminating between the two presented directions of the moving dots. Our previous study confirmed that anodal tDCS over the left hMT+ improved motion direction identification in noisy conditions through signal enhancement or noise reduction (Wu et al., 2020). Additionally, anodal tDCS over V1 led to an improvement in visual orientation discrimination (Sczesny-Kaiser et al., 2016). This evidence suggests that the hMT+ and V1 may be participate in the identification of direction against noise and the discrimination of the direction, respectively. In current study, participants trained on a condition with 100% coherence, a task in which identification of the motion direction could not be improved by applying tDCS over the hMT+, since there were no randomly moving dots. That is, the learning task used here included two cognitive processes, but there was a ceiling effect on direction identification because of the high coherence (100%) in the training task. Thus, tDCS over the hMT+ did not improve direction identification. Nevertheless, the other cognitive process (discrimination between two directions of motion; related to V1) was not assessed in this study. In sum, this study did not find an effect of tDCS on learning to discriminate the direction of motion of 100% coherent dots. Thus, future research should use other motion perception tasks in which coherence is variable.

Second, the current study aimed to detect learning enhancement over multiple sessions, rather than a simple within-session change in behavior. Studies have shown that tDCS over the left hMT+ can directly improve visual motion perception measured during or immediately after stimulation (Battaglini et al., 2017; Wu et al., 2020). However, VPL often requires thousands of trials over multiple sessions to improve (Herpich et al., 2019). Thus, VPL involves complex cognitive processes such as sleep-mediated consolidation (He et al., 2021, 2022; Yang et al., 2022). To date, it remains unknown whether tDCS facilitates VPL across multiple sessions. Consistent with our results, several studies did not find a significant influence of tDCS on multisession VPL (Fertonani et al., 2011; Larcombe et al., 2018).

Third, the timing of stimulation and training may be important. Pirulli et al. (2013) found that anodal tDCS facilitated task performance if it was applied before training, whereas transcranial random noise stimulation (tRNS) had a facilitated effect only when it was applied during training. The authors suggested that tDCS induced neuronal depolarization mainly through the initiation of homeostatic mechanisms. These homeostatic mechanisms might not be totally functional if engaged during a task but will eventually induce stronger aftereffects. Therefore, anodal tDCS has effects that last after the end of stimulation, and performance improvement is more likely if tDCS is applied before the execution of training. Conversely, other studies found that online application of tDCS (during training) led to greater improvements in performance on tasks involving motor learning (Nitsche et al., 2003; Stagg and Nitsche, 2011) and VPL (Sczesny-Kaiser et al., 2016; Karlaftis et al., 2021). The influence of timing on VPL merits further study.

We found a stronger sensation induced by anodal tDCS compared with sham condition across all training sessions. Indeed, many previous studies also reported a difference in subjective feelings between active and sham tDCS (Ambrus et al., 2010; Larcombe et al., 2018). Obvious sensation induced by active tDCS may make participants pay more attention to the execution of task. However, we think there is no such possibility given the between-subjects design of this study. Participants in one group were only subject to one type of tDCS throughout the entire experiment; they therefore did not experience different feelings and cannot judge whether a stimulation is real or a sham stimulation.

Magnetic resonance spectroscopy (MRS) provides a noninvasive imaging technique to measure changes in cortical neurotransmitter concentrations from within a defined region of interest. It has been found that various neurotransmitter systems (GABA, Glutamate, dopamine, serotonin, etc.) may all have an impact on tDCS effect (Mclaren et al., 2018). For example, anodal tDCS has been shown to be excitatory, result in decreased GABA levels in visual (Barron et al., 2016), and facilitate visual learning (Frangou et al., 2018; Karlaftis et al., 2021). Additionally, anodal tDCS reduces local GABA while cathodal stimulation reduces glutamatergic neuronal activity with a highly correlated reduction in GABA (Stagg et al., 2009). Although this study did not find the significant effect of tDCS on VPL according to the behavioral results, the neural mechanisms should be further explored.

As mentioned above, the effect of tDCS on VPL is inconsistent in previous studies. Variability in tDCS effects have resulted in calling for greatly increased sample sizes (Minarik et al., 2016). Our sample size (*n* = 9 per group) is comparable to or greater than several tDCS studies in the VPL that found significant effects (Antal et al., 2004a; Herpich et al., 2019) although it is smaller than more studies (Sczesny-Kaiser et al., 2016; Karlaftis et al., 2021). The null effects in our study do not exclude the possibility of a smaller effect that could be detected with a larger sample size.

In conclusion, although this study failed to find a significant effect of tDCS on motion direction discrimination learning, it is too early to conclude that tDCS does not affect VPL, since the results thus far are inconsistent. Thus, further study of this topic is needed. Three possibilities should be considered during future research: task selection, number of training sessions and the timing of stimulation.

## Data availability statement

The raw data supporting the conclusions of this article will be made available by the authors, without undue reservation.

## Ethics statement

The studies involving human participants were reviewed and approved by Air Force Medical University. The patients/participants provided their written informed consent to participate in this study.

## Author contributions

DW and YW completed the experiment and wrote the manuscript. DW and NL assisted with the experiment and analyzed the data. KS and PW provided technical guidance and site support. WX conceived the idea and provided financial support. All authors contributed to the article and approved the submitted version.
